# Improving the Youth HIV Prevention and Care Continuums: The Adolescent Medicine Trials Network for HIV/AIDS Interventions

**DOI:** 10.2196/12050

**Published:** 2019-03-26

**Authors:** Sonia Lee, Bill G Kapogiannis, Susannah Allison

**Affiliations:** 1 Eunice Kennedy Shriver National Institute of Child Health and Human Development Bethesda, MD United States; 2 National Institute of Mental Health Bethesda, MD United States

**Keywords:** HIV, youth, adolescent, treatment, care continuum

## Abstract

**Background:**

Epidemiologic and clinical information in the United States indicate that HIV transmission and acquisition among adolescents and young adults (youth) remain unchanged, without improvement. Interventions to prevent HIV transmission among youth are critically needed, as are interventions to improve adherence to all components of the continuum of care for youth living with HIV.

**Objective:**

The primary mission of the Adolescent Medicine Trials Network for HIV/AIDS Interventions (ATN) is to conduct both independent and collaborative research that explores promising behavioral, microbicidal, prophylactic, therapeutic, and vaccine modalities in HIV-infected and at-risk youth aged between 12 and 24.

**Methods:**

Through the ATN, the National Institutes of Health is supporting HIV interventional research for youth in the United States.

**Results:**

The ATN comprises 3 cooperative multiproject research programs and a coordinating center. Each program is led by a network hub and has well-defined research themes to assist, guide, and coordinate HIV research project activities.

**Conclusions:**

ATN activities encompass the full spectrum of research needs for youth, from HIV primary prevention for at-risk youth in the community to secondary and tertiary prevention with clinical management of HIV infection among youth living with HIV experiencing adherence challenges.

## Introduction

### Background

Epidemiologic and clinical information in the United States indicate that HIV transmission and acquisition among adolescents and young adults (youth) remain unchanged, without improvement. The Centers for Disease Control and Prevention (CDC) estimates that in the United States, youth aged 13 to 24 years accounted for 21% of all new HIV diagnoses in 2016 [1]. In 2016 alone, among the approximately 8450 youth diagnosed with HIV in the United States, 80.99% (6844/8450) were gay or bisexual males. The CDC also estimates that at the end of 2015, an estimated 60,300 youth were living with HIV in the United States [2]. Of these, 51.00% (30,753/60,300) were living with undiagnosed HIV—the highest rate of undiagnosed HIV in any age group. Increased efforts for routine HIV testing have not substantially increased the numbers of diagnoses among youth who are unaware that they are living with HIV infection. To obtain a better understanding of the transmission risks and needs of youth to improve HIV testing and diagnosis rates, systematized and widespread monitoring and surveillance of outcomes along the entire HIV prevention continuum of care, including linkage to prevention services and engagement in risk-reduction interventions, are needed [3-6]. Furthermore, interventions to prevent HIV transmission among youth are critically needed, including behavioral interventions and studies of HIV vaccines, microbicides, and pre-exposure prophylaxis (PrEP) uptake and adherence.

Not only do youth and young adults have the highest rates of undiagnosed HIV [1] but they also have some of the poorest outcomes across the HIV treatment and care cascade [7-9]. They have the lowest rate of linkage to care (among youth who were diagnosed with HIV in 2014, 68% were linked to care within 1 month) and the lowest rate of viral suppression for any age group (among youth who were diagnosed with HIV in 2012 or earlier, 55% were retained in HIV care and 44% had a suppressed viral load) [1]. Linkage to care is a critical step for youth who test HIV-positive that is hampered by barriers on many levels, including structural, individual, and developmental [10]. Numerous studies have documented the challenges that youth experience maintaining adherence to their antiretroviral regimens, including side effects, medication dosing schedules, unstable living situations, disclosure of HIV status, co-occurring illnesses, and lack of health insurance [11-13]. Recent data, however, suggest that youth can be successfully treated at health care sites that have expertise in treating youth [14], whereby 59% of youth achieved sustained viral load suppression over the course of a year. Health care delivery and other interventions tailored to the unique needs of youth and their effects on HIV continuum of care outcomes need to be investigated further [15]. Studies are urgently needed to better understand the factors leading to poor outcomes for youth across the care continuum and to identify strategies that can substantially improve the achievement of essential milestones along the care continuum, ultimately helping infected youth achieve durable viral suppression. Given the adherence challenges that many youth experience, trials are needed to study newer drug schedules and formulations that allow simpler regimens, evaluation of programs to promote antiretroviral treatment adherence in youth, and clinical trials to evaluate therapies that may exploit the immunologic resilience of recently infected youth.

### Objectives

Through the Adolescent Medicine Trials Network for HIV/AIDS Interventions (ATN), the National Institutes of Health (NIH) is supporting HIV interventional research for youth in the United States. The primary mission of the ATN is to conduct both independent and collaborative research that explores promising behavioral, microbicidal, prophylactic, therapeutic, and vaccine modalities in HIV-infected and at-risk youth aged between 12 and 24. ATN activities encompass the full spectrum of research needs for youth, from HIV primary prevention for at-risk youth in the community to secondary and tertiary prevention with clinical management of HIV infection among youth living with HIV experiencing adherence challenges. Primary prevention research addresses motivational readiness for and subsequent uptake of biomedical prevention strategies, including innovative, technology-based interventions tailored for youth at greatest risk for HIV infection or transmission. Secondary and tertiary prevention research investigates novel treatment strategies and regimens, antiretroviral therapy (ART) adherence, risk reduction interventions, and linkage to and long-term engagement in care strategies that can lead to optimal ART initiation and sustained virologic suppression. Innovative strategies to engage youth are especially important in vulnerable populations such as youth, who may encounter more obstacles and challenges when attempting to access care [10,15]. Furthermore, as reflective of the US epidemic, a significant portion of the current ATN portfolio of studies seeks to enroll traditionally difficult-to-reach populations of medically disenfranchised, low socioeconomic status, sexual and gender minority, and/or racial or ethnic minority young men and women.

## Methods

### Adolescent Medicine Trials Network for HIV/AIDS Interventions 2001-2013

The ATN is the only domestic, multicenter research network devoted to the health and well-being of HIV-positive and at-risk youth. The NIH initiated the ATN in 2001 after the Adolescent Medicine HIV/AIDS Research Network external scientific advisory panel stated that interventional studies in adolescents were needed. The first, second, and third funding cycles ended in February 2006, 2011, and 2016, respectively; the ATN was re-competed in 2016 and was funded for a fourth, 5-year period. The ATN has demonstrated extensive experience in recruiting and retaining understudied at-risk and HIV-infected youth populations in the United States. During the 10-year period (2003-2013), more than 26,000 youth have been enrolled among 88 ATN studies, with >90% enrollment and retention rates among completed studies. The ATN published descriptive findings of 1712 youth living with HIV/AIDS, recruited from December 2009 to January 2011 from 15 ATN sites to participate in a cross-sectional survey of demographic, psychosocial, and health factors [16]. From 1 of the largest national samples of adolescents and young adults living with HIV, distinct patterns of risk behaviors were identified, pointing to the importance of tailoring clinical and preventive interventions. A more recent longitudinal cohort study at 14 ATN sites across the United States also reported demographic and psychosocial characteristics of 467 youth living with HIV and assessed how the later steps of the HIV continuum of care were achieved over a 1-year period [14]. Among 32 studies with completed analyses, the ATN has published over 214 manuscripts and presented over 156 abstracts at various domestic and international scientific conferences. In addition, the ATN has successfully forged collaborations with other federal agencies and NIH-funded HIV research networks, as evidenced by 12 coendorsed collaborating protocols.

### Adolescent Medicine Trials Network for HIV/AIDS Interventions 2016-2021

**Figure 1:**
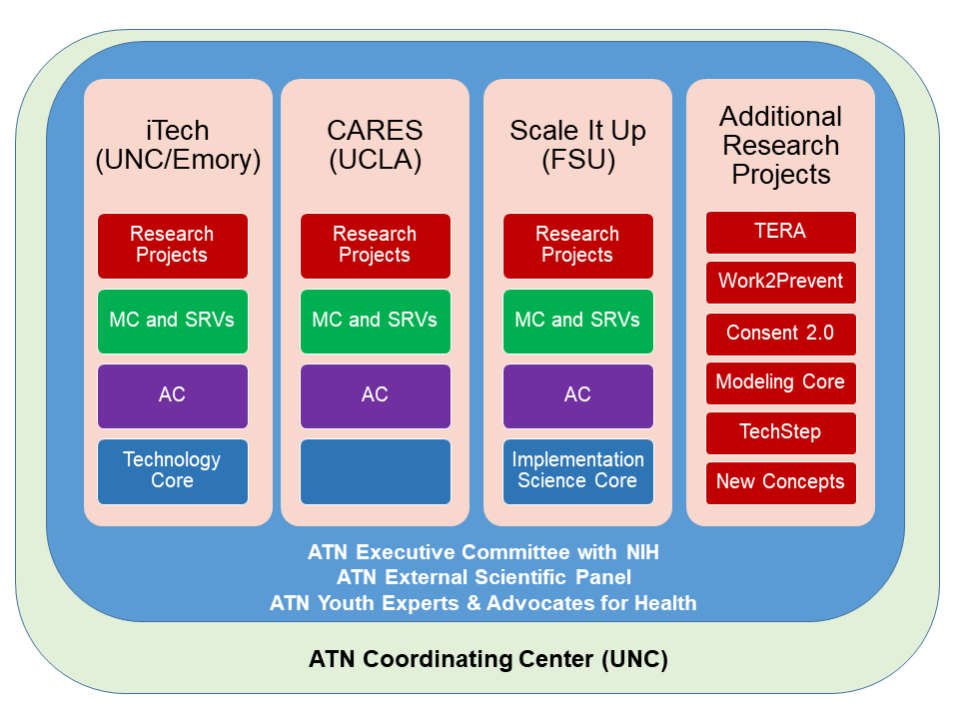
Adolescent Medicine Trials Network for HIV/AIDS Interventions structure. AC: Analytic Core; CARES: Comprehensive Adolescent Research & Engagement Studies; FSU: Florida State University; iTech: University of North Carolina/Emory Center for Innovative Technology; MC: Management Core; NIH: National Institutes of Health; SRVs: Subject Recruitment Venues; TERA: Triggered Escalating Real-time Adherence; UCLA: University of California Los Angeles; UNC: University of North Carolina.

In 2016, the ATN was newly structured to better align network resources with scientific priorities and further increase collaborations both within the ATN and with other HIV research networks through 3 adolescent-focused HIV/AIDS clinical trial network hubs (Figure 1).

Led by the *Eunice Kennedy Shriver* National Institute of Child Health and Human Development, the ATN is cofunded by the National Institute on Drug Abuse, the National Institute of Mental Health, and the National Institute on Minority Health and Health Disparities, through 3 cooperative multiproject research programs. Each program is coordinated and led by a network hub and has well-defined research themes, with substantial NIH scientific involvement to assist, guide, and coordinate research project activities. The research projects are resourced with a Management Core for the overall management, coordination, and scientific oversight of the program. Projects are also resourced with an Analytic Core to ensure that shared scientific and analytic resources are available alongside the methodologic and analytic expertise necessary to evaluate their scientific progress. An optional resource for each program is a Scientific Core with unique resources and expertise to support the success of the research. The new structure is designed to create transparent mechanisms for network investigative leaders to solicit and support ideas from the research community and allow for external researchers as well as other networks to benefit from the networks’ infrastructure and capacity, and support coordinated efforts with community partners.

## Results

### Adolescent Medicine Trials Network for HIV/AIDS Interventions Multiproject Research Programs

The principal investigators and the programs they lead are summarized in Table 1, with research project titles provided in Table 2. Detailed protocol information regarding each of the ongoing research projects have been published separately [17], with results from the projects expected at the end of the ATN 5-year cycle in 2021. The ATN Comprehensive Adolescent Research & Engagement Studies (CARES) Program will evaluate community-based strategies to leverage gateways and settings where high-risk and youth living with HIV can be engaged in HIV prevention and treatment in 2 HIV epicenters (Los Angeles, CA, and New Orleans, LA). The ATN University of North Carolina/Emory Center for Innovative Technology (iTech) Program aims to lower the burden of HIV infection by developing and evaluating innovative, interdisciplinary research on technology-based interventions across the HIV prevention and care continuum for at-risk or youth living with HIV. The ATN Scale It Up Program specifically focuses on developing, testing, and bringing to practice self-management interventions that positively impact the youth HIV prevention and care cascades. Furthermore, efforts to refine the ATN research agenda will be augmented by an External Scientific Panel, a group of members with a wide range of expertise, who will review the scientific progress and activities of and provide input to the ATN, with the goal of maintaining the highest level of scientific productivity, quality, and relevance of proposed and ongoing research projects.

### Adolescent Medicine Trials Network for HIV/AIDS Interventions Governance

Overall, the integration of efforts across the programs is overseen by an ATN Executive Committee (EC) through leadership, efficient communication, coordination, and scientific collaboration. The ATN EC also facilitates collaborations with other HIV research networks and investigators, maintaining the overall responsibility for developing, implementing, and adapting the clinical research agenda of the ATN to include the following:

1. Primary prevention interventions
  - Biomedical prevention interventions
  - Novel approaches to identifying undiagnosed infection
  - Behavioral and social interventions (eg, multilevel, combination prevention, mental health, substance use studies) to address uptake of HIV prevention strategies
2. HIV continuum of care
  - Interventions and programs, both independent and collaborative, to improve outcomes
  - Community- and structural-level interventions to improve outcomes
  - Strategies to address evolving health care financing challenges
  - Evaluation of long-acting antiretroviral therapy for treatment
  - Risk-reduction interventions
  - Interventions to promote care engagement and adherence to antiretroviral medications
  - Integrated treatment approaches (psychological, medical, and ancillary services studies).

Furthermore, the EC oversees the coordination and scientific collaboration of the ATN’s National Community Advisory Board, self-titled Youth Experts and Advocates for Health (ATN-YEAH). The ATN-YEAH comprises 12 youth representatives from the programs’ local CABs and provides expertise, consultation, and perspective to ensure that the research agenda and work of the ATN reflects and addresses the current needs and issues of youth. Members also provide feedback on recruitment materials, ATN Network policies, research study protocols and procedures, and potential collaborations. They also serve as liaisons to local ATN-related Community Advisory Boards. Logistically, the members meet via virtual meetings on a quarterly basis and via an annual face-to-face meeting to collaborate on community HIV prevention and care activities, learn from each other’s experiences regarding how HIV affects their lives, and provide input on different research project updates. The annual meeting occurs in conjunction with 1 of the biannual ATN meetings where an ATN-YEAH panel session is highlighted to foster open dialogue between the ATN-YEAH members and ATN research investigators regarding HIV research topics and issues. Youth perspectives and input to the ATN research agenda are further augmented by the direct participation of 3 youth representatives on the EC itself, with voting rights.

The vital importance of stimulating the engagement of junior investigators with fresh perspectives, additional bandwidth, and innovative ideas to the success of the ATN’s research has spurred the ATN to prioritize the mentorship of junior-level investigators through a mentorship/scholars initiative, also monitored by the EC. This initiative includes a comprehensive, research career development program for scholars who come from communities most affected by HIV/AIDS in the United States and who are underrepresented in the scientific field. The program aims to help scholars acquire the skills and expertise to develop and sustain productive, rewarding youth-focused HIV research careers; successfully compete for independent research grants; and develop collaborative working relationships within the context of the ATN’s research efforts.

**Table 1:** Adolescent Medicine Trials Network for HIV/AIDS Interventions multiproject research programs.

Adolescent Medicine Trials Network for HIV/AIDS Interventions multiproject research program	Principal investigators	Principal investigator’s institution	Research theme	Number of research projects
Comprehensive Adolescent Research & Engagement Studies	MJ Rotheram-Borus, PhD	University of California, Los Angeles	Comprehensive, community-based strategies for youth recruitment and engagement	3
iTech	L Hightow-Weidman, MD, MPH, and P Sullivan, PhD, DVM	University of North Carolina, Chapel Hill, and Emory University	Innovative, interdisciplinary, technology-based interventions	10
Scale It Up	S Naar, PhD	Florida State University	Effectiveness-implementation research to enhance youth self-management	4

**Table 2:** Adolescent Medicine Trials Network for HIV/AIDS Interventions research projects.

ATN^a^ protocol number	ATN research program	ATN project title
ATN 138	iTech	YouThrive: Connecting Youth and Young Adults to Optimize ART^b^ Adherence Through the Interactive YouTHrive WebApp
ATN 139	iTech	Get Connected: Linking YMSM^c^ to Adequate Care through a Multilevel, Tailored WebApp Intervention
ATN 140	iTech	LYNX: A Novel Mobile App to Support Linkage to HIV/STI^d^ Testing and PrEP^e^ for YMSM
ATN 141	iTech	MyChoices: Mobile-Based Application to Increase Uptake of HIV Testing, Detection of New HIV Infections, and Linkage to Care and Prevention Services by Young Men who have Sex with Men
ATN 142	iTech	P3: Prepared, Protected, emPowered: Promoting PrEP Adherence through a Social Networking, Gamification, and Adherence Support App for Men and Transgender Women Who Have Sex With Men
ATN 143	iTech	Compare: Comparing Efficacy of LYNX (ATN 140) and MyChoices (ATN 141) Mobile Applications for HIV Testing and PrEP Uptake
ATN 144	Scale It Up	SMART: Adaptive Antiretroviral Therapy Adherence Interventions for Youth Living with HIV through Text Messaging and Cell Phone Support Embedded within the Sequential Multiple Assignment Randomized Trial (SMART) Design
ATN 145	Scale It Up	Young Men’s Health Project: Comparative Effectiveness Trial of Clinic-Based Delivery of an HIV Risk Reduction Intervention for YMSM
ATN 146	Scale It Up	TMI: Tailored Motivational Interviewing Implementation Intervention Effectiveness Trial in Multidisciplinary Adolescent HIV Care Settings
ATN 147	CARES^f^	Acute, Recent and Established Youth Living with HIV
ATN 148	CARES	Stepped Care for Youth Living with HIV: Optimizing the HIV Treatment Continuum with a Stepped Care Model for Youth Living with HIV
ATN 149	CARES	Cost-efficient Interventions for Youth at Risk for HIV: Engaging Seronegative Youth to Optimize the HIV Prevention Continuum
ATN 150	ATN Coordinating Center	Consent 2.0: Innovative Approaches for Minor Consent to Biomedical HIV Prevention Research
ATN 151	ATN Coordinating Center	Work2Prevent: Employment as HIV prevention for Young Men who have Sex with Men (YMSM) and Young Transgender Women (YTW)
ATN 152	ATN Coordinating Center	TERA: A Triggered, Escalating, Real-Time Adherence Intervention
ATN 155	ATN Coordinating Center	Planning4PrEP: Integrating PrEP into Family Planning Services at Title X Clinics in the Southeast
ATN 156	Scale It Up	WeTest: Enhancing sexual safety: Couples’ communication and HIV testing among YMSM
ATN 157	iTech	We Prevent: A Relationships Skills Intervention to Improve HIV Prevention Uptake Among Young Gay, Bisexual and other Men who have Sex with Men and their Primary Partners
ATN 158	iTech	Life Steps for PrEP for Youth (LSPY) An Evidence-based Cognitive Behavioral Adherence Intervention to Enhance PrEP Uptake and Adherence in High Risk YMSM
ATN 159	iTech	ePrEP: A Randomized, Controlled Trial of an Electronic HIV Pre-exposure Prophylaxis Care System Among Young Men who have Sex with Men in Rural and Small Town Areas
ATN 160	iTech	TechStep: Technology‐based Stepped Care to Stem Transgender Adolescent Risk Transmission
ATN 161	ATN Coordinating Center	ATN Modeling Core: Investing in the HIV care continuum: Model‐based methods to translate ATN findings into policy recommendations

^a^ATN: Adolescent Medicine Trials Network for HIV/AIDS Interventions.

^b^ART: antiretroviral therapy.

^c^YMSM: young men who have sex with men.

^d^STI: sexually transmitted infection.

^e^PrEP: pre-exposure prophylaxis.

^f^CARES: Comprehensive Adolescent Research & Engagement Studies.

The ATN also encompasses a coordinating center (CC at the University of North Carolina, Chapel Hill, with PIs L LaVange, PhD, M Carpenter, PhD, and M Hudgens, PhD) to provide overall infrastructure and logistical and organizational support for the ATN and to facilitate emerging studies and collaborative activities across the ATN and with external networks and investigators. Support for these newly emerging activities includes statistical, data management, study management, study quality assurance, and operational services and infrastructure. The CC also administratively manages a suite of high-priority research protocols (see Table 2) addressing issues such as adolescent consent for research, employment for transgender youth, ART adherence, integration of HIV prevention with family planning, and a modeling core that uses innovative, model-based methods to translate findings from ATN studies into policy recommendations.

## Discussion

The ATN aims to change the sobering trends among youth affected by HIV in the United States through innovative approaches and a suite of research projects that address many high-priority scientific questions. Overall, the objectives and overarching goals of the ATN are to increase the number of at-risk youth who are aware of their HIV status and bend the infection rate curve downward toward zero, and for those who are diagnosed with HIV, to increase the numbers in each segment of the care continuum to 95%. The ATN with its highly experienced, multidisciplinary investigators has a renewed focus to address the HIV epidemic by addressing both individual and structural issues particularly salient to adolescents. These include stigma, substance use, mental health difficulties, developmental challenges and transitions to adulthood, and availability of and access to youth-friendly health services. Important additional priorities include facilitating mentorship of junior-level investigators as well as including the voices of diverse youth input throughout all activities of the ATN. The ATN remains committed to performing the highest priority research and disseminating findings in a timely and transparent manner with the primary goal of ending the youth HIV epidemic in the United States.

## Abbreviations

ART: antiretroviral therapy
ATN: Adolescent Medicine Trials Network for HIV/AIDS Interventions
CC: coordinating center
CDC: Centers for Disease Control and Prevention
EC: Executive Committee
iTech: University of North Carolina/Emory Center for Innovative Technology
NIH: National Institutes of Health
PrEP: Pre-Exposure Prophylaxis
YEAH: Youth Experts and Advocates for Health
YMSM: young men who have sex with men
